# Satellite Observations and Malaria: New Opportunities for Research and Applications

**DOI:** 10.1016/j.pt.2021.03.003

**Published:** 2021-03-25

**Authors:** Michael C. Wimberly, Kirsten M. de Beurs, Tatiana V. Loboda, William K. Pan

**Affiliations:** 1 Department of Geography and Environmental Sustainability, University of Oklahoma, Norman, OK, USA; 2 Department of Geographical Sciences, University of Maryland, College Park, MD, USA; 3 Duke Global Health Institute, Duke University, Durham, NC, USA

## Abstract

Satellite remote sensing provides a wealth of information about environmental factors that influence malaria transmission cycles and human populations at risk. Long-term observations facilitate analysis of climate–malaria relationships, and high-resolution data can be used to assess the effects of agriculture, urbanization, deforestation, and water management on malaria. New sources of very-high-resolution satellite imagery and synthetic aperture radar data will increase the precision and frequency of observations. Cloud computing platforms for remote sensing data combined with analysis-ready datasets and high-level data products have made satellite remote sensing more accessible to nonspecialists. Further collaboration between the malaria and remote sensing communities is needed to develop and implement useful geospatial data products that will support global efforts toward malaria control, elimination, and eradication.

## Satellite Observations and Malaria

Since 2000, considerable progress has been made in reducing the global burden of malaria, shrinking the malaria map, and moving toward the goal of malaria eradication [1–3]. However, there is concern that declines in malaria cases and deaths have slowed [4]. Although the reasons for this slowdown are multifaceted, an important factor is the limited set of tools and approaches that are currently available for combating malaria. The recent Lancet Commission report on malaria eradication emphasized that new technologies, including innovations in the field of malaria informatics, are needed to facilitate more effective data-driven management of malaria interventions [5]. Geospatial data, including satellite observations, were highlighted as a key data source for monitoring human populations and their environments in support of malaria eradication. This recommendation is concordant with other assessments that have emphasized the importance of spatial decision support systems to enable national and subnational program management as well as regional and global strategic planning [6].

**Figure 1.**
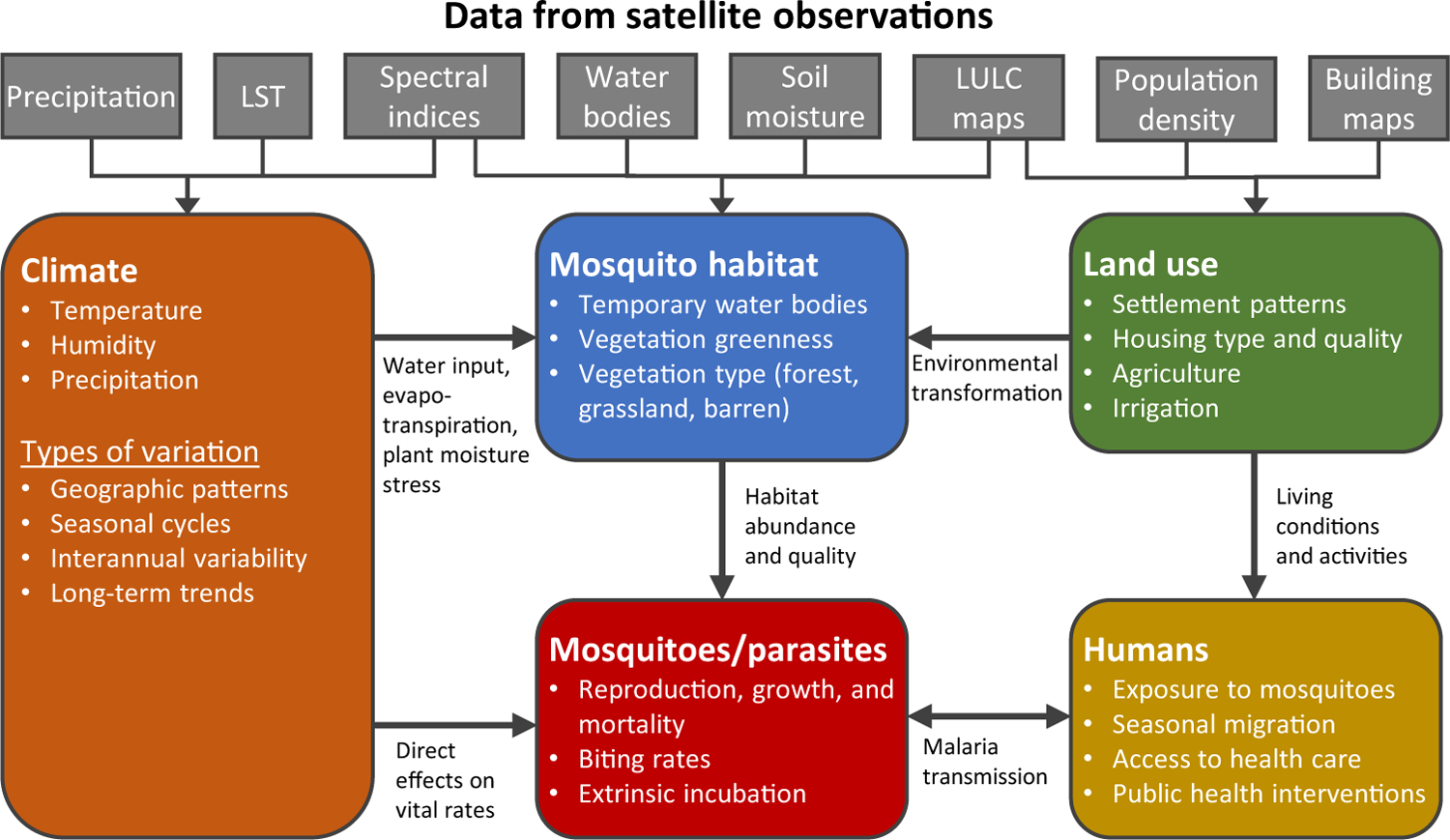
Pathways through Which Satellite Data Provide Information about Malaria. Satellite data can be used to predict geographic patterns and changes over time in climate factors, mosquito habitats, and human land use. These environmental variables influence malaria transmission through their effects on mosquitoes, parasites, and humans. Abbreviations: LST, land surface temperature; LULC, land use and land cover.

**Figure 2.**
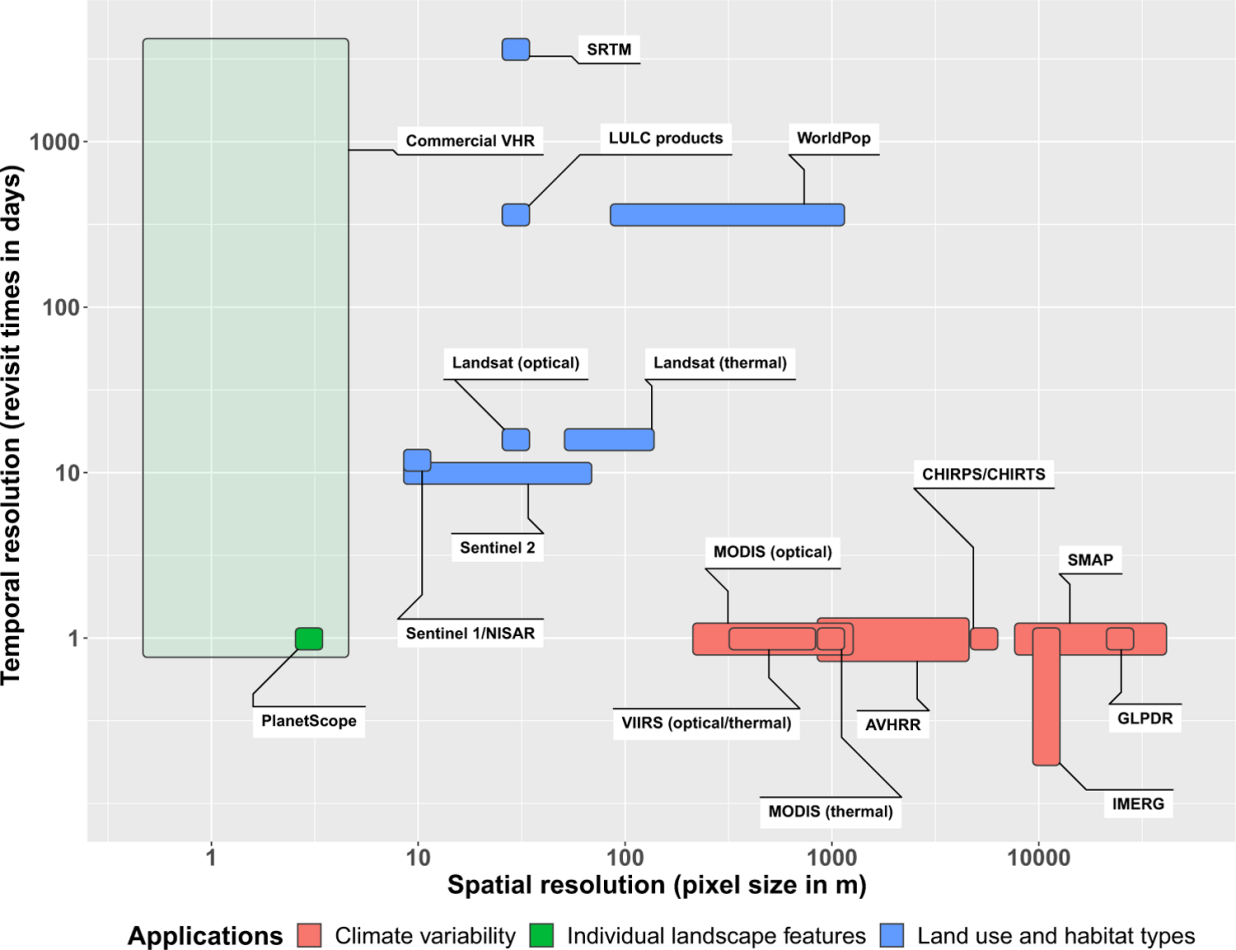
Spatial and Temporal Resolutions of Satellite Missions and Data Products with Applications to Malaria. Colors represent the main applications of satellite data at different resolutions. The lighter green color of the Commercial VHR box indicates the variable frequency of image acquisition. In principle, most VHR commercial satellites can collect data on a daily or near-daily repeat cycle. In practice, these satellites image only a fraction of the Earth’s surface each day, acquisition strategies are based on demand from customers, and remeasurement frequency can range from days to years. Abbreviations: AVHRR, advanced very-high-resolution radiometer; CHIRPS/CHIRTS, climate hazards group infrared precipitation/temperature with stations; GLPDR, global land parameter data record; IMERG, integrated multisatellite retrievals for global precipitation measurement; LULC, land use and land cover; MODIS, moderate resolution imaging spectroradiometer; NISAR, NASA-ISRO synthetic aperture radar; SMAP, soil moisture active-passive; SRTM, shuttle radar topography mission; VHR, very high resolution; VIIRS, visible infrared imaging radiometer suite.

The value of satellite observations for malaria research has long been recognized, with the earliest reviews appearing more than two decades ago [7–9]. Since then, considerable changes in global antimalaria efforts and advances in the field of remote sensing have taken place. Myriad connections between the environmental phenomena observed by satellite-borne sensors and different aspects of the malaria transmission cycle have been identified (Figure 1). However, the challenges of discovering, accessing, and processing relevant satellite data (Figure 2) still limit their use for malaria projects. The purpose of this review is to present an up-to-date assessment of satellite missions relevant to malaria and identify opportunities where new sources of remote sensing data can be leveraged to support novel applications. Major themes include long-term satellite records of environmental changes that affect malaria risk, new sources of satellite data with higher spatial resolution, measurement frequency, and global coverage, and emerging technologies that can increase the accessibility and usability of remote sensing data in the malaria sector.

## Long-term Records of Environmental Change

Malaria transmission cycles are sensitive to climate variability, and satellite observations provide accurate, reliable, and timely information about these variations. Precipitation influences the hydrological cycles of aquatic habitats for anopheline larvae [10], while temperature and humidity affect the vital rates that drive mosquito population dynamics, parasite development in the mosquito, and parasite transmission [11]. Climate also affects malaria indirectly through influences on land use, settlement patterns, and human population movements [12]. The densities of *in situ* weather stations, as well as the quality and completeness of the data collected, are limited in many of the low- and middle-income countries where malaria is a public health concern. Therefore, satellite data are an important information source for characterizing multidecadal trends and monitoring ongoing changes in these areas. Relevant satellite measurements include precipitation estimates, **land surface temperature** (see Glossary), and **spectral indices** like the normalized difference vegetation index (NDVI) that are sensitive to vegetation and moisture (Box 1).

To assess relationships between climate variations and malaria, it is essential to have long-term records combined with frequent measurements to capture short-term anomalies and seasonal cycles (Figure 2). Early applications of remote sensing for malaria research relied on NDVI and land surface temperature measured by the advanced very-high-resolution radiometer (AVHRR) instrument on United States National Oceanic and Atmospheric Administration (NOAA) weather satellites. AVHRR provides daily, global observations dating back to 1981 at a nominal resolution of 1000–4000 m [8]. The moderate resolution imaging spectroradiometer (MODIS) instrument, launched aboard the United States National Aeronautics and Space Administration (NASA) Terra and Aqua satellites in 1999 and 2002, provided significant improvements in spatial resolution (250–1000 m), measurement frequency (up to four times daily in the tropics), number of **spectral bands**, and data quality.

**Figure 3.**
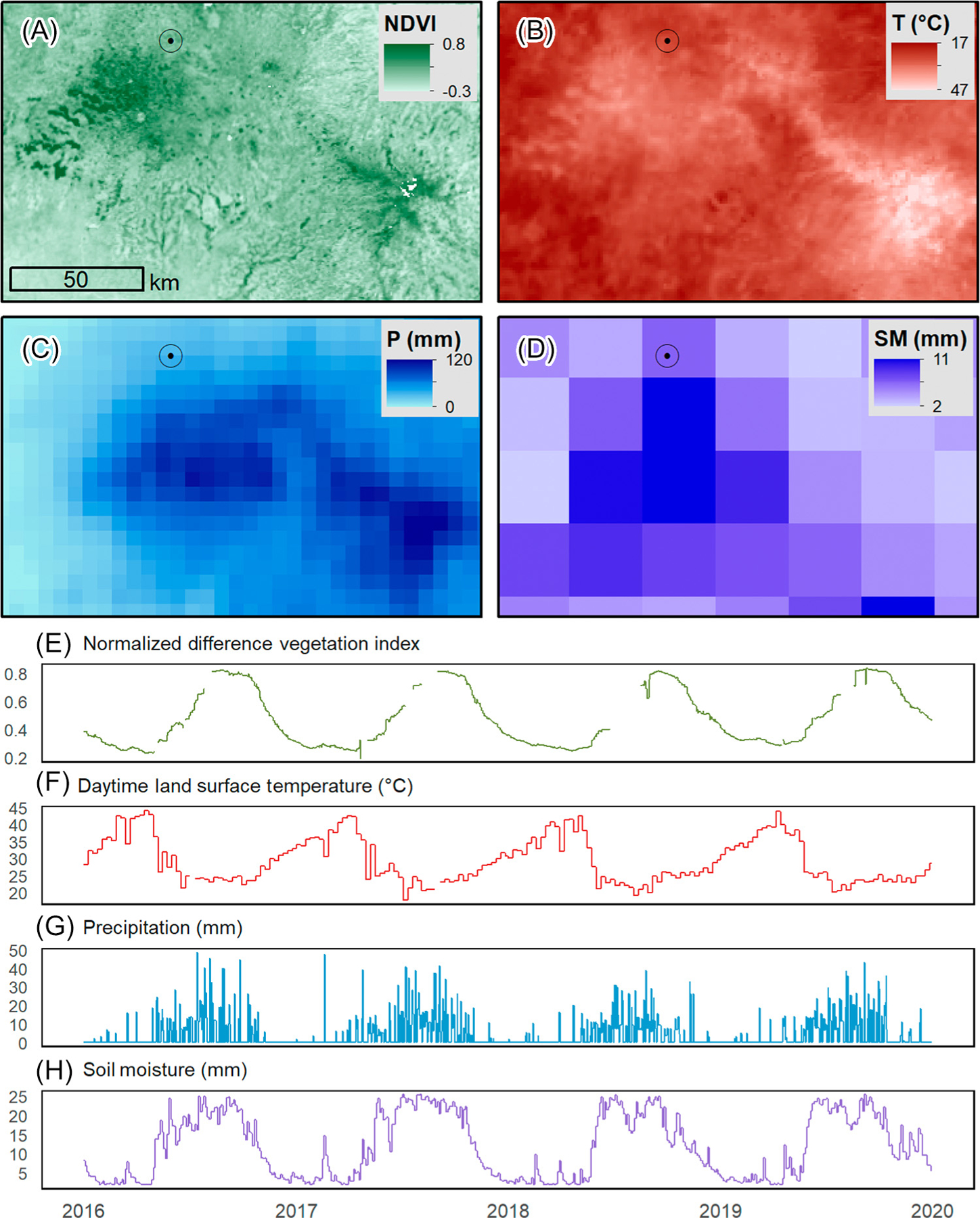
Satellite Data Collected over the Choke Mountains of Northwest Ethiopia in March 2019. The four images display (A) moderate resolution imaging spectroradiometer (MODIS) normalized difference vegetation index (NDVI) on 1 March 2019; (B) MODIS daytime land surface temperature (LST) on 1 March 2019; (C) climate hazards group infrared precipitation with station (CHIRPS) total monthly precipitation from 1 March to 31 March 2019; and (D) soil moisture active passive (SMAP) soil moisture on 1 March 2019. The time series charts display 4 years of data collected at the highlighted point on the maps. (E) MODIS NDVI. (F) MODIS LST. (G) CHIRPS precipitation. (H) SMAP soil moisture. The geographic coordinates of the highlighted point are 11.292 N, 36.978 E.

Spectral indices and land surface temperature from MODIS are frequently used with satellite precipitation measurements (Figure 3) as predictors in spatial models for generating malaria risk maps [13,14], and time series models for predicting changes in malaria risk resulting from environmental fluctuations [15,16]. Remotely sensed data are also used to control for environmental variation when studying the influences of other factors on malaria. A study of the effects of malaria interventions in Africa used MODIS data to control for climate variation and night-lights data from the newer visible infrared imaging radiometer suite (VIIRS) to control for urbanization [2,17]. Similarly, an analysis of cross-border malaria spillover in the Amazon used meteorological and hydrological variables derived from satellite observations to control for variation in environmental risk factors [18].

Despite the demonstrated value of MODIS for malaria research, the spatial resolution is unsuitable for mapping finer-grained landscape details. Land use practices and the resulting land cover patterns can increase or decrease the abundance of mosquitoes and their potential to transmit malaria depending on social, ecological, and geographic contexts. Irrigated agriculture can provide larval habitats for anopheline mosquitoes and is a risk factor for malaria in many, but not all, settings [19]. Urbanization increases malaria risk in parts of Asia where the primary vector is the urban-adapted *Anopheles stephensi* [20] but reduces malaria risk in Africa where vectors such as *Anopheles gambiae* are associated with rural habitats [21]. Forest cover is a risk factor for malaria in parts of Southeast Asia where the vector *Anopheles dirus* is associated with closed-canopy forests [22]. In contrast, deforestation can increase habitat suitability for vector species such as *An. gambiae* s.l. in Africa and *Nyssorhynchus darlingi* (formerly *Anopheles darlingi*) in South America, leading to higher malaria risk in cleared areas [23,24]. Human activities associated with land use practices, including agriculture and forest work, also influence exposure to bites of infected mosquitoes [25,26].

**Figure 4.**
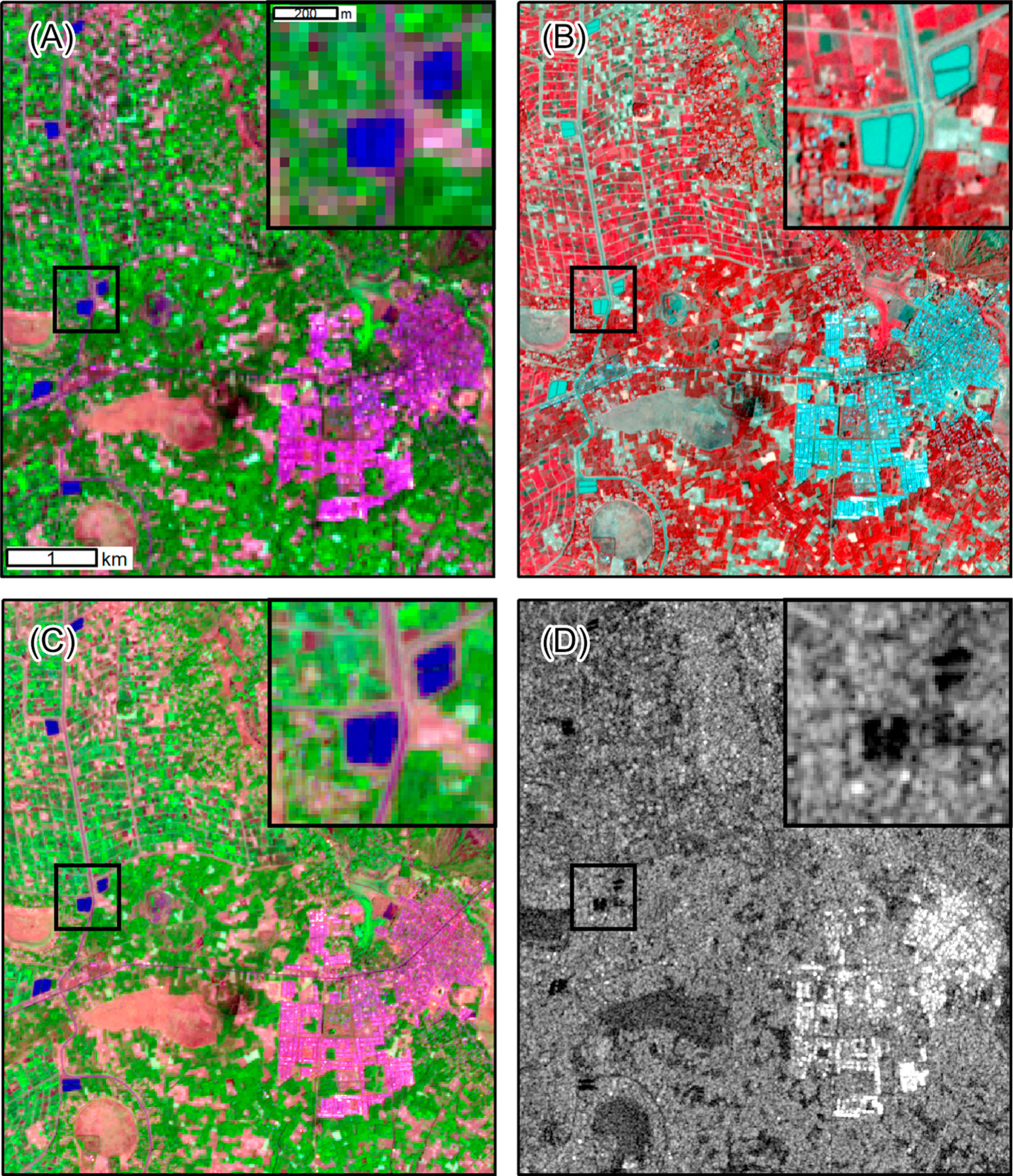
Satellite Images of a Landscape in the Mecha District of Ethiopia in March 2019. There is irrigated agriculture in the northwest part of the maps and the town of Merawi is located in the southeast. The zoomed inset map (dark boxes) shows irrigation canals and water storage ponds. (A) Landsat 8 false-color composite (shortwave infrared band displayed as red, near-infrared band displayed as green, and green band displayed as blue). Vegetation appears green, impervious surfaces are pink and purple, wetlands are reddish-brown, and open water is dark blue. (B) PlanetScope false-color composite (near infrared band displayed as red, red band displayed as green, green band displayed as blue). Vegetation appears red, impervious surfaces are white and light blue, wetlands are gray, and open water is light blue. (C) Sentinel-2 false color composite. Band display and interpretation are the same as for Landsat. (D) Sentinel-1 synthetic aperture radar image displaying the strength of signal returned to the sensor. The brightest areas indicate strong returns from buildings. Darker areas indicate open water and wet areas that reflect the signal away from the sensor. The geographic coordinates of Merawi are 11.410 N, 37.154 E.

To measure these landscape features with precision, higher-resolution data from satellite missions such as Landsat are needed (Figure 4A). The Landsat program has collected optical data at 30 m resolution and thermal data at 60–120 m resolutions since the launch of Landsat 4 in 1982 (Figure 2). Although the 16-day revisit time of a Landsat satellite is considerably longer than the daily resolution of MODIS, it is suitable for measuring change in land cover at seasonal to annual time scales. Most applications of Landsat for malaria risk assessment have used one or a few images to provide a static assessment of landscape conditions at a single point in time. Examples include the influences of vegetation and water on spatial patterns of malaria cases in Ethiopia [27], Swaziland [28], and the Brazilian Amazon [29], and high-resolution malaria risk mapping in Vietnam [30] and Madagascar [31]. Landsat is also frequently used to create classified land cover maps and derive vegetation and moisture indices for analysis and mapping of anopheline mosquito habitats [23,32,33].

All new and historical Landsat data became freely available in 2010, catalyzing advances in remote-sensing science that have enabled high-resolution global land cover mapping at annual time steps [34]. Landsat-derived annual measurements of forest gain and loss from a global dataset [35] were used to study the effects of forest clearing and fragmentation on the occurrence of *Plasmodium knowlesi* in humans in Malaysian Borneo [36]. The Program to Calculate Deforestation in the Amazon (PRODES) dataset, which uses Landsat and other data sources to map annual deforestation in the Brazilian Legal Amazon, has been used to examine the effects of forest loss on malaria incidence in this region [37–39]. A 30-year global dataset of surface water was incorporated into a map of malaria vector suitability in Malawi [40], and a 14-year time series of Landsat-derived NDVI was used in a model of malaria cases at the health facility level in Zambia [41]. There is potential for much broader use of Landsat time series in malaria research. One limiting factor is the availability of long-term epidemiological and entomological datasets with high enough spatial resolution to associate with land use and land cover changes. Working with Landsat time series is also technically challenging because of large data volumes and data gaps resulting from cloud cover.

Data continuity is an important issue for remote-sensing applications. All satellite missions have a finite lifespan, and differences in sensor and orbital characteristics affect the measurements taken by newer missions. Harmonized products can be developed to combine satellite data from different sources into consistent, long-term datasets. An example is the suite of Integrated Multi-Satellite Retrievals for GPM (IMERG) products, which provide seamless precipitation estimates from 2000 to the present by combining data from the current Global Precipitation Measurement (GPM) mission, the older Tropical Rainfall Measurement Mission (TRMM), and other sources. The resulting data record has been used in the study of malaria and other water-related diseases [42]. A significant event for the remote-sensing community will be the end of the MODIS era, with the decommissioning of Terra expected in 2026 and Aqua several years afterwards. MODIS is being replaced by the VIIRS sensor onboard NOAA polar-orbiting satellites, and there is ongoing research on harmonizing MODIS and VIIRS to generate consistent records of land surface temperature and vegetation indices [43,44]. However, it is not yet clear what types of harmonized data products will be available and how accessible and usable these data will be for work with malaria and other environmentally sensitive diseases.

## New Spatial, Temporal, and Spectral Resolutions

Household-level research and interventions require information about individual dwellings and community interventions that target high-resolution spatial features like ponds and temporary water bodies. Mapping this level of detail requires very high resolution (VHR) satellite imagery (ranging from <1 m to 5 m pixel size), which is acquired by commercial satellites such as Ikonos, GeoEye, and WorldView 1–3. These images can be used to enumerate individual households for selecting study subjects or implementing household-level interventions [45–47]. Building data obtained from VHR imagery are also useful for developing localized population estimates in settings where national census data are unavailable or inadequate [48]. Individual water bodies that serve as larval habitats are also detectable in VHR images, and this information can be used to map areas with high mosquito abundance and target mosquito control activities [49,50]. However, because of persistent cloud cover during rainy seasons it is often difficult to acquire VHR images at the times when larval habitats are most abundant.

A considerable amount of VHR imagery is publicly available via online platforms such as Google Earth, Google Maps, and Bing Maps. These images are compressed true-color (red, green, and blue) composites that are suitable for visual interpretation [51] and some digital processing and classification techniques [52]. Imagery dates vary, although most are within the past several years. With Google Earth, it is possible to determine imagery dates and also access historical VHR data. In principle, most VHR commercial satellites can collect data on a daily or near-daily repeat cycle. In practice, these satellite images cover only a fraction of the Earth’s surface each day, acquisition strategies are based on demand from customers, and historical data coverage is highly variable in space and time (Figure 2).

The raw satellite data used to generate these public images offer more opportunities for analysis and usually include one or more infrared bands that are useful for detecting water bodies and healthy vegetation [53,54]. However, the high cost and technical challenges of working with commercial VHR data have limited its broader use for malaria research and applications. In addition to the price of image acquisition, manipulating and analyzing large volumes of VHR imagery requires considerable storage space and processing power. VHR data are often classified using object-based methods that identify and classify spatial clusters of pixels rather than the pixel-based approaches used with coarser-resolution imagery [55,56]. These techniques require specialized software and expertise that are not available to most end users in the malaria sector.

An important advance has been the development of ‘smallsats’ – compact and relatively inexpensive satellites that can be produced and deployed in large numbers. The PlanetScope mission, which began in 2016, consists of more than 120 smallsats that collect daily 3 m resolution VHR satellite data for the entire globe (Figure 4B). Unlike other sources of VHR satellite data, with PlanetScope it is feasible to track seasonal and interannual environmental variability. The high temporal resolution also results in a higher probability of obtaining cloud-free data for a particular location and time period. Specific applications have included tracking human rights violations [57], identifying croplands affected by plant diseases [58], and monitoring spatial and temporal patterns of small water bodies in arid regions [59]. PlanetScope data should also be well suited for tracking short-term changes in larval habitats and the extent and condition of human settlements.

The Sentinel-2 mission, launched by the European Space Agency (ESA) in 2015, likewise has a global data acquisition strategy with images acquired weekly at spatial resolutions ranging from 10 to 20 m (Figure 4C). Although these resolutions are too coarse for detecting individual larval habitats or dwellings, Sentinel-2 can be used to map land cover characteristics such as irrigated agriculture, urban areas, and water at a finer spatial resolution than is possible with Landsat. Compared to PlanetScope, Sentinel-2 data are free and have higher radiometric quality as well as middle-infrared bands that are useful for mapping vegetation and water. There have been several promising applications of Sentinel-2 for mapping water bodies and mosquito-breeding habitats [60–62]. Data from Sentinel-2 have also been combined with data from Landsat 8 to develop a harmonized 30 m product that greatly increases the frequency of observations [63].

Most work on remote sensing of malaria has used data from **passive sensors** that detect reflected and emitted energy in the optical and thermal wavelengths (Box 1). In contrast, **synthetic aperture radar** (SAR) is an **active sensor** that emits pulses of microwave radiation and measures the energy returned to the sensor (Box 2). SAR has been used to map landscape features such as irrigated agriculture, open water, and wetlands that provide habitats for mosquito larvae [64,65]. Most importantly, SAR instruments collect data at longer wavelengths that can penetrate substantial cloud cover, but a major constraint on the use of SAR for malaria applications has been data availability. In the past, SAR data were mostly collected by commercial satellites and were relatively expensive, while the smaller amount of free SAR data had limited spatial and temporal coverage. The launch of the first Sentinel-1 satellite by the ESA in 2014 initiated a new era of free global access to SAR data (Figure 4D). Sentinel-1 provides 10 m resolution C-band SAR data for the entire globe on a 12-day repeat cycle. Sentinel-1 has been used to map open water and wetlands that provide larval habitats for malaria vectors in Zambia [66] and the Amazon [67]. The upcoming NASA-ISRO SAR (NISAR) mission, scheduled for launch in 2022, will provide an additional global source of free L- and S-band SAR data. In general, end users in the public health sector are less familiar with radar than optical and thermal remote sensing. However, with increasing availability of free SAR data, more training resources are becoming available to support their broader use [68].

Passive microwave sensors detect emitted microwave radiation (Box 2). These data can be used to estimate soil moisture and may be useful for identifying saturated soils where water is likely to pool and create larval habitats. The Soil Moisture Active-Passive (SMAP) mission, launched in 2015, generates daily soil moisture data at 9 and 36 km resolutions (Figure 3). The ESA Climate Change Initiative soil moisture product provides a long-term record of soil moisture from 1978 to the present based on data from multiple active and passive microwave sensors at a spatial resolution of ~28 km [69]. The Global Land Parameter Data Record (GLPDR), which is derived from passive microwave observations, includes daily observations, from 2002 to 2018, of soil moisture along with fractional water cover, air temperature, and atmospheric water vapor [70]. A study of mosquito populations in the USA found that these remotely sensed variables were more strongly associated with mosquito abundance than were meteorological variables from local weather stations [71]. To date, passive microwave data have not been widely used for malaria applications. Although the coarse spatial resolution limits their use for local assessments, there is potential for incorporating soil moisture data at broader regional and global scales.

In addition to these new sources of satellite data, the emergence of other sensor platforms, such as drones, is an important development [72]. Although drones are less suitable than satellites for regular long-term monitoring they have the advantage of being deployable at the specific times and locations desired by the user. Other relevant technologies include inexpensive data loggers that can be used to monitor microclimates for mosquito vectors across many sites [73] and location technologies, such as global positioning system (GPS) trackers and mobile phones, that can monitor the movements of people and parasites [74]. There is much potential for integrating these technologies with satellite remote sensing, such as using drones to collect very-high-resolution data on larval habitats and training satellite image classifiers to extrapolate predictions over larger areas and longer time periods.

## New Technology to Enhance Data Accessibility and Usability

Although many satellite datasets are available online at no cost (see Table S1 in the supplemental information online), accessing them requires navigating a vast and often confusing array of data products (Figure 2), determining which are suitable for a particular application, and downloading large volumes of data from online archives. Multiple processing steps are usually necessary, including data extraction from complex archive files, reprojection to match the coordinate systems of other geospatial datasets, computing environmental indices from raw data, detecting and filling data gaps, and summarizing the results at appropriate temporal and spatial units [75]. Most malaria researchers and practitioners lack experience with satellite data processing and do not have access to the specialized software that is needed. One way to handle this challenge is by assembling interdisciplinary teams of scientists and practitioners that can work collaboratively to connect satellite remote sensing with malaria. More generally, there is a need to enable data sharing across different disciplines by developing tools to facilitate data retrieval and implementing policies that provide access to both epidemiological and remote sensing data for research and applications.

The organizations that provide satellite data, such as the NASA Earth Observing System Data and Information System (EOSDIS) in the USA, provide tools and services to enable data discovery and downloading. Although many of these tools are targeted to remote sensing experts, others have been developed to streamline access for users in other disciplines. The Giovanni system developed by the Goddard Earth Sciences Data and Information Services Center provides a web-based interface that facilitates data search, visualization, and download [76]. The International Research Institute for Climate and Society’s data library [77] includes a simplified online interface for manipulating, visualizing, and downloading climate data, including climate-based maps of malaria risk [78]. Remote sensing data are increasingly being distributed as ‘analysis-ready’ datasets or ‘data cubes’ where preprocessed data products are provided as gridded tiles that can be incorporated into analytical workflows with minimal additional processing [79–81]. These systems incorporate user-friendly interfaces for browsing data as well as application programming interfaces (APIs) that can automate large data downloads. As new tools are implemented to enhance data discovery, accessibility, and usability, the barriers to working with remote sensing data will continue to decline.

Another important trend is the increasing availability of higher-level products that use satellite data to map land cover and meteorological variables at regional to global extents. Global land cover datasets include forest cover [35], surface water [82], and urban areas [83] at 30 m resolution. Satellite-derived land cover data are also an important input into high-resolution population maps such as those produced by WorldPop [84]. High-resolution global meteorological datasets include the Climate Hazards Group Infrared Precipitation with Stations (CHIRPS) and Climate Hazards Group Infrared Temperature with Stations (CHIRTS) which provide daily precipitation and air temperature data at a spatial resolution of ~5.5 km [85,86]. These products can improve malaria–climate research by providing more localized estimates of the meteorological conditions influencing mosquitoes and malaria transmission than lower-resolution meteorological grids. The datasets have typically undergone some accuracy assessment, but data quality can vary greatly from location to location. Therefore, it is strongly recommended to assess the suitability of these global products before applying them for local and regional malaria assessments.

New **cloud computing** environments for remote sensing data analysis have also facilitated broader use of remote sensing data. Google Earth Engine (GEE) includes a browser-based interactive development environment and a JavaScript application programming interface that provide access to a wide range of satellite products [87]. Computations are carried out via parallel processing in the Google Cloud, facilitating analysis over large areas and long time periods. The cloud-based implementation also allows access for end users with limited computational resources in low-bandwidth environments. To date, the use of GEE for public health applications, and malaria in particular, has been limited [88,89]. However, recent studies have used GEE to map mosquito habitats in Malawi [40] and other countries in southern Africa [33]. Commercial computing service providers, such as Google Cloud and Amazon Web Services, have also provided access to extensive satellite data archives via their platforms. Other emerging platforms for cloud-based analysis of Earth observation datasets that could be useful for malaria applications include Sentinel Hub, Open Data Cube (ODC), and the System for Earth Observation Data Access, Processing and Analysis for Land Monitoring (SEPAL) [90].

## Concluding Remarks

Satellite remote sensing is now routinely used in malaria research to measure environmental conditions that influence mosquito populations, human vulnerability, and malaria transmission cycles. These relationships provide the basis for risk maps that highlight locations with the highest malaria risk [31,41] and early warning systems that forecast malaria outbreaks based on lagged responses to environmental variation [91]. Satellite data can also be used to map buildings, estimate human population density, and identify land use practices that affect human exposure to mosquitoes. An important goal is to incorporate this information into spatial decision support systems that target malaria interventions at the locations and times when they will be most effective [6]. Despite the prospects, satellite observations are still not routinely incorporated into malaria decision support, and there are few published studies focused on operational use. To alleviate this gap, there is a need to develop and test new tools that use remotely sensed data for specific malaria applications. Implementation research is also needed to evaluate the impacts of these tools on malaria programs and determine how they can be used more widely and effectively to support malaria control and elimination (see Outstanding Questions).

The development of novel malaria applications is facilitated by the growing availability of satellite imagery and corresponding increases in the spatial and temporal resolution of these data. Although VHR data are essential for identifying individual landscape features like buildings and small waterbodies, this level of detail is not necessary for all applications. For example, to map mosquito-breeding habitats created by irrigation, classifying irrigated agriculture using free, 30 m resolution Landsat data can be just as effective and much more efficient than attempting to identify every water body with <1 m imagery. In general, data with coarser spatial resolution have higher temporal resolution, which allows closer tracking of changing environmental conditions (Figure 2). Determining the best approaches for particular problems will require more research on the spatial and temporal scales at which environmental change influences malaria transmission.

In addition to malaria, satellite observations can be applied to a broader array of parasitic diseases, particularly those with arthropod vectors or zoonotic hosts such as leishmaniasis, lymphatic filariasis, human African trypanosomiasis, and schistosomiasis. Remote sensing has also been widely used for research on other mosquito-borne diseases, including those caused by arboviruses such as dengue, Zika, and West Nile virus. Because of the differences in vector ecology and transmission cycles, the specific approaches will vary for different diseases. However, efforts to improve the accessibility and usability of remote sensing data for malaria will have the added benefit of increasing opportunities to use these data for other public health applications. The malaria and remote-sensing communities should work collaboratively to identify the characteristics of remote-sensing data that make them most useful for malaria and other public health applications. This information can guide efforts to synthesize the ever-expanding archive of satellite observations into more accessible and useful products that will support global efforts toward malaria control, elimination, and eradication.

## Supplementary Material

Supp.Materials

## Highlights

Long-term satellite records supply data on environmental variables that influence malaria transmission cycles.

High-resolution land use and land cover maps from satellite observations provide information about human activities that affect mosquito habitats and exposure to mosquito bites.

New sources of very-high-resolution satellite data create opportunities for precise, localized mapping of mosquito habitat and human settlements.

Global availability of free synthetic aperture radar data facilitates mapping of buildings, water, and land use in cloudy conditions that are characteristic of many tropical regions.

New cloud-based technologies for remote sensing data access, processing, and analysis are lowering the barriers to data use for malaria applications.

## Outstanding Questions

What characteristics of satellite remote-sensing data are most useful for malaria research and applications?

What are the best approaches for making satellite data accessible to, and useable by, scientists conducting malaria research and practitioners involved in malaria programs?

How can new global sources of remote-sensing data, including commercial very-high-resolution imagery and synthetic aperture radar data, be applied to produce new information for malaria decision support?

How can satellite earth observations be used most effectively to enhance spatial decision support systems and improve malaria program management?

## Box 1. Optical and Thermal Remote Sensing

Sensors on Earth-observing satellites detect energy within one of more spectral bands. Each band captures radiation that is reflected or emitted by different objects on the Earth’s surface within a rather narrow range of electromagnetic radiation. Bands in the visible portion of the spectrum detect blue (~400 nm), green (~500 nm), and red (~700 nm) light, while bands in the near-infrared (750–1000 nm) and shortwave-infrared (1200–2500 nm) portions of the spectrum have varying sensitivities to vegetation, water, and soils. Measurements of **radiance** taken by the sensors are transformed into **reflectance**, which measures the fraction of incoming solar radiation reflected from the Earth’s surface back to the sensor.

Reflectance data are commonly used to compute spectral indices. The NDVI is calculated from near-infrared and red bands and is sensitive to healthy green vegetation. The normalized difference moisture index (NDMI) is calculated from near-infrared and shortwave-infrared bands and is sensitive to vegetation moisture stress. Because spatial and temporal variation in vegetation tracks fluctuations in temperature and precipitation, these indices provide an indirect measurement of underlying environmental patterns that influence mosquitoes and malaria. Other spectral indices like the normalized difference water index (NDWI), calculated from green and near-infrared bands, can detect open water bodies that provide larval habitats. Depending on the sensor, these indices can be mapped at spatial resolutions ranging from less than a meter to thousands of meters.

Thermal sensors detect emitted radiation from the thermal infrared portion of the spectrum (3–14 μm). Measurements of radiance in the thermal wavelengths can be used to estimate land surface temperature, which measures how much heat radiates from the uppermost part of the Earth’s surface. This surface may be a grass lawn, bare soil, the roof of a building, or the leaves at the top of a forest. Mosquitoes are directly influenced by air temperature, which is generally correlated with land surface temperature. During the day, near-surface air temperature often deviates from land surface temperature because of the effects of solar radiation, wind, and soil moisture. During the night, solar radiation does not influence land surface temperature and the correlation with air temperature is usually stronger than during the day. Because of these differences, land surface temperature is most useful as a relative indicator of temperature variation in space and time when used as an environmental indicator of malaria risk.

## Box 2. Microwave Remote Sensing

**Synthetic aperture radar (SAR)** is an active remote sensing method that uses microwave energy with wavelengths ranging from a few centimeters up to a meter. Wavelengths commonly used for terrestrial remote sensing include X-band (2.4–3.8 cm), C-band (3.8–7.5 cm), and L-band (15–30 cm). The land surface affects the backscatter of these microwaves depending on its roughness, geometry, and electrical characteristics, influencing the strength of the signal returned to the sensor. Shorter wavelengths are sensitive to foliage and branches at the tops of vegetation canopies, whereas longer wavelengths can penetrate vegetation canopies and detect characteristics of the land surface underneath. The resulting data can be used to identify land cover features relevant to malaria, including water, soil, vegetation, and buildings. SAR is also used to measure terrain, including the shuttle radar topography mission (SRTM) dataset which is an important global source of high-resolution (30 m) elevation data.

In contrast to radar, passive microwave sensors detect energy emitted from the Earth’s surface. Because the energy level of emitted microwave radiation is very low, it must be measured over large areas. Therefore, passive microwave data typically has a spatial resolution of 10 km or more. Like backscattered microwaves from radar, passive microwaves are sensitive to land surface properties such as roughness, vegetation, and moisture. Passive microwaves are commonly used to detect soil moisture, which is relevant for malaria because saturated soils are likely to form pools that can serve as larval habitats. Passive microwaves are also used in meteorological remote sensing to develop satellite-based precipitation estimates.

A major advantage of active and passive microwave remote sensing is their low sensitivity to cloud cover. Compared to optical and thermal remote sensing, microwave remote sensing has far fewer data gaps resulting from clouds. This characteristic is valuable for malaria applications as the tropical areas where malaria is a persistent public health threat tend to have high cloud cover, particularly during monsoon seasons.

## Glossary

Active sensor: measures signals transmitted by the sensor that are reflected, refracted, or scattered by the Earth’s surface or atmosphere
Cloud computing: the delivery of remote computing services, including processors, storage, and software, as virtualized resources over the internet
Land surface temperature: the amount of heat radiating from the uppermost layer of the Earth’s surface
Passive sensor: measures reflected solar energy or emitted energy from the Earth’s surface
Radiance: the flux density of radiant energy measured by a sensor, measured in watts per steridian per square meter per meter
Reflectance: the amount of radiation measured by a sensor expressed as a proportion of the incoming solar radiation
Spectral band: a specific range of wavelengths over which electromagnetic radiation is measured by a sensor
Spectral index: an index that is calculated as a function of two or more spectral bands to measure vegetation greenness, vegetation moisture, or other characteristics of the Earth’s surface
Synthetic aperture radar (SAR): an active remote sensing technique for generating high-resolution radar images by taking advantage of the movement of the sensor to create a very large ‘virtual’ antenna
