# Supplementary material for: Satellite Observations and Malaria: New Opportunities for Research and Applications
Source: Trends Parasitol. Author manuscript; Available in PMC 2021 Jun 1. (PMC8122067; doi:10.1016/j.pt.2021.03.003)
Supplement: Supp.Materials [file NIHMS1683221-supplement-Supp_Materials.docx]

Supplementary file

**Satellite Observations and Malaria: New Opportunities for Research and Applications**

Michael C. Wimberly^1^*, Kirsten M. de Beurs^1^, Tatiana V. Loboda^2^, William K. Pan^3^

1 Department of Geography and Environmental Sustainability, University of Oklahoma, Norman OK, USA.

2 Department of Geographical Sciences, University of Maryland, College Park MD, USA.

3 Duke Global Health Institute, Duke University, Durham, NC, USA.

*Correspondence: [mcwimberly@ou.edu](mailto:mcwimberly@ou.edu) (M. C. Wimberly)

**Table S1. List of satellite missions and data products mentioned in the article.**

| **Satellite Mission/Product** | **Years** | **Spatial Resolution (m)** | **Repeat Time** | **Type** | **Notes** | **Sources** |
| --- | --- | --- | --- | --- | --- | --- |
| Climate Hazards Group Coupled Infrared Precipitation with Stations (CHIRPS) | 1981-present | ~5,500 | 1 day | Rainfall Estimate | Incorporates satellite and meteorological station data | <https://www.chc.ucsb.edu/data/chirps> |
| Climate Hazards Group Coupled Infrared Temperature with Stations (CHIRTS) | 1983-present | ~5,500 | 1 day | Air Temperature Estimate | Incorporates satellite and meteorological station data | <https://chc.ucsb.edu/data/chirtsdaily> |
| Global Artificial Impervious Area | 1985-2018 | 30 | 1 year | Impervious Surface Estimates | Derived from Landsat data | <http://data.ess.tsinghua.edu.cn> |
| Global Forest Change | 2000-present | 30 | 1 year | Forest Cover and Change Estimates | Derived from Landsat data | <https://earthenginepartners.appspot.com/science-2013-global-forest/download_v1.7.html> |
| Global Surface Water | 1984-present | 30 | 1 year | Water Cover and Change Estimates | Derived from Landsat data | <https://global-surface-water.appspot.com> |
| Integrated Multi-Satellite Retrievals for Global Precipitation Measurement (IMERG) | 2000-present | ~11,000 | 30 minutes | Rainfall Estimate | Harmonized product using data from multiple missions | <https://gpm.nasa.gov/data/directory> |
| Land Parameter Data Record | 2002-2018 | 25,000 | 1 day | Soil Moisture, Air Temperature, Water Cover, Water Vapor | Derived from passive microwave data | <https://nsidc.org/data/NSIDC-0451> |
| Landsat (TM, ETM+, OLI, and TIRS) | 1984-present | 30 (optical-IR), 60-120 (thermal) | 16 days | Optical, Near-IR, Shortwave-IR, Thermal | Two satellites (Landsat 7 and 8) currently active with Landsat 9 planned for 2021 | <https://www.usgs.gov/core-science-systems/nli/landsat/landsat-science-products> |
| PlanetScope | 2017-present | 3 | 1 day | Optical, Near-IR | Commercial, constellation of microsatellites | <https://www.planet.com> |
| Sentinel-1 | 2014-present | 10 | 12 days | C-band radar | Two satellites currently active | <https://asf.alaska.edu/data-sets/sar-data-sets/sentinel-1/sentinel-1-data-and-imagery/> |
| Sentinel-2 | 2015-present | 10-60 | 10 days | Optical, Near-IR, Shortwave-IR | Two satellites currently active | <https://www.usgs.gov/centers/eros/science/usgs-eros-archive-sentinel-2> |
| Soil Moisture Active Passive (SMAP) | 2015-present | 9,000-36,000 | 1 day | Soil Moisture Estimate | Derived from passive microwave data | <https://nsidc.org/data/smap/smap-data.html> |
| Shuttle Radar Topography Mission (SRTM) | 2000 | 30 | NA | Gridded elevation data | Derived from C- and X-band SAR | <https://www.usgs.gov/centers/eros/science/usgs-eros-archive-digital-elevation-shuttle-radar-topography-mission-srtm-1-arc> |
| Suomi NPP (VIIRS) | 2011-present | 375-750 | 1 day | Optical, Near-IR, Shortwave-IR, Thermal | Follow-on to MODIS, Afternoon overpass (1:30 PM) | <https://viirsland.gsfc.nasa.gov/index.html> |
| Terra/Aqua (MODIS) | 2001-present | 250-1000 | 1 day | Optical, Near-IR, Shortwave-IR, Thermal | Two satellites (Terra with 10:30 AM overpass, Aqua with 1:30 PM overpass) | <https://modis.gsfc.nasa.gov/data/dataprod/> |
| WorldPop | 2000-present | 100-1,000 | 1 year | Population Estimates | Derived from census data and satellite imagery | <https://www.worldpop.org> |
| WorldView | 2009-present | 0.5-2 |  | Optical, Near-IR, Shortwave-IR | Commercial, series of four satellites | <https://www.satimagingcorp.com> |

ETM+: enhanced thematic mapper; IR; infrared; MODIS: moderate-resolution imaging spectroradiometer; NPP: national polar-orbiting partnership; OLI: operational land imager; TM: thematic mapper; TIRS: thermal infrared sensor; VIIRS: visible infrared imaging radiometer suite.
